# A protocol to execute a lab-on-chip platform for simultaneous culture and electrochemical detection of bacteria

**DOI:** 10.1016/j.xpro.2023.102327

**Published:** 2023-05-26

**Authors:** Sonal Fande, Sangam Srikanth, Jayapiriya U S, Khairunnisa Amreen, Satish Kumar Dubey, Arshad Javed, Sanket Goel

**Affiliations:** 1 Department of Electrical and Electronics Engineering, Birla Institute of Technology and Science (BITS) Pilani, Hyderabad Campus, Hyderabad 500078, India; 2 Department of Mechanical Engineering, Birla Institute of Technology and Science (BITS) Pilani, Hyderabad Campus, Hyderabad 500078, India; 3 MEMS, Microfluidics and Nanoelectronic (MMNE) Lab, Birla Institute of Technology and Science (BITS) Pilani, Hyderabad Campus, Hyderabad 500078, India

**Keywords:** Biotechnology and bioengineering, Material sciences

## Abstract

Here, we present a protocol for a miniaturized microfluidic device that enables quantitative tracking of bacterial growth. We describe steps for fabricating a screen-printed electrode, a laser-induced graphene heater, and a microfluidic device with its integrations. We then detail the electrochemical detection of bacteria using a microfluidic fuel cell. The laser-induced graphene heater provides the temperature for the bacterial culture, and metabolic activity is recognized using a bacterial fuel cell.

Please see Srikanth et al.^1^ for comprehensive information on the application and execution of this protocol.

## Highlights

• Simultaneous bacterial culture and detection using a microfluidic device
• Electrochemical analysis through screen-printed electrode modified with GMC
• Obtain bacteria concentration in the range of 2 × 10^4^ to 1.1 × 10^9^ CFU/ml
• Fabricate portable lab-on-a-chip platform by 3D packaging

## Abstract (editor highlights)

Publisher’s note: Undertaking any experimental protocol requires adherence to local institutional guidelines for laboratory safety and ethics.

## Graphical abstract

**Figure:**
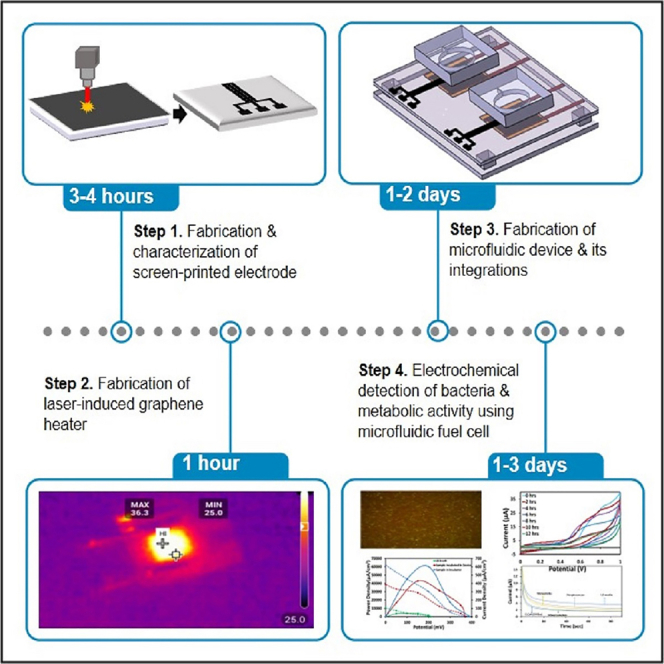

## Before you begin

Microbial detection is crucial in numerous fields, for example, medical research, food technologies, and biochemical detection.^2^ The traditional methods, such as colony counting, gram-staining, and another biochemical way like Enzyme linked immunoassay (ELISA), Polymerase chain reaction (PCR) are time-consuming,^3^ show transfer errors, and require more labor to count microbial cells in a particular solution. Hence, a valid and sensitive approach to detecting bacteria is critical.^4^ Here, a simple, economical, and competent way is used to detect bacteria. The electrochemical analysis^5^ is carried out to check the growth of bacteria in intervals of every 2 h up to 12 h, which will be helpful for the early diagnosis of bacterial infection.

The protocol below describes a stepwise procedure for developing a microfluidic-based electrochemical platform for simultaneous cultivation and bacterial growth detection.^6^ Here we use the electrochemical method to monitor the growth of bacteria. The bacterial culture with LB broth media is injected into the microfluidic chamber. The same is kept in the conventional incubator every 2 h, of the cyclic voltammetry response is recorded. And the result obtained is compared with a traditional incubator.

### Materials

**Timing: 1–2 h (for step 4)**

**Timing: 1–2days (for step 5)**

1. Multi-walled carbon nanotubes (MWCNT) are acquired from Sigma Aldrich, India, and conductive carbon ink is purchased from Engineered Materials System, Inc. The supplier of Ag/AgCl is ALS Co. Ltd. in Japan.
2. Poly (methyl methacrylate) is obtained from Dali Electronics, India. A CO_2_ laser (VLS 3.60) is procured from Universal Laser Systems, Arizona, USA. The thermal camera is purchased from Fluke Technologies, India.
3. Potassium Ferricyanide is purchased from AVRA chemicals. Polydimethylsiloxane (PDMS) is purchased from Dow Corning, USA. Luria Bertani is purchased from SRL Chemicals.
4. Preparation of a three-electrode system
  a. Prepare a reference electrode by modifying it with Ag/AgCl.
  b. Prepare a working electrode by modifying it with MWCNT.
    i. Directly drop-cast MWCNT over the electrode surface.
    ii. Leave it to dry at room temperature (20°C–25°C) for 20 min.
    iii. Prepare the counter electrode with plane carbon ink (without any modification).
5. Preparation of bacterial culture medium

This step describes how to prepare a bacterial culture medium.

The Luria Bertani (LB) is the medium employed in this protocol. 5 g of LB powder and 250 mL of distilled water are mixed to prepare LB media.

6. To prepare the bacterium culture,^7^ mix 0.2 mL of cultured E. coli bacteria with 20 mL LB media.
7. Keep the sample intact in a CO2 incubator with an orbital shaker, and adjust the temperature and speed to 36°C and 180 RPM, respectively.

***Note:*** The CO2 incubator provides a stable atmosphere with the following parameters: 37°C, and relative humidity of roughly 95%, which help microbial cell growth.

8. Check OD value every 2 h.

***Note:*** The culture can be utilized for the experiment once the desired OD (0.8–1) is reached.

9. Next, use the agar plating (Pour-plate) method to detect the colony-forming units. Prepare the agar gel by dissolving 8.7 gm of agar powder in 250 mL of water.
10. Autoclave the dissolved agar solution at 15 psi 120°c for 20 min.
11. Transfer the sterilized autoclaved agar solution into the Petri plates until the agar gel gets solidified.
12. Spread over the 10 μL of over the plate, and keep the containers in the incubator for 24 h at 37°c.

***Note:*** Sterile condition is required throughout the bacterial culture process.

## Key resources table

**Table:** 

REAGENT or RESOURCE	SOURCE	IDENTIFIER
**Bacterial strains**		

Luria Bertani	SRL Chemicals	29817
*E. coli* strain	Department of Biology, BITS Pilani Hyderabad campus	-
Agar medium	SRL Chemicals	L3147

**Chemicals**		

Conductive carbon ink	Engineered Materials System	CI-2001
Ag/AgCl	ALS Co. Ltd., Japan	011464
Multi walled carbon nanotubes (MWCNT)	Sigma-Aldrich	308068-56-6
Polyimide	Dali Electronics	150FNB019
Potassium ferricyanide	AVRA Chemicals	13746-66-2
PDMS and curing agent	Dow Corning, USA	101697

**Software and algorithms**		

AutoCAD	Autodesk	https://getintopc.com/?s=AutoCad

**Other**		

CO_2_ laser	Universal Laser Systems, AZ, USA	VLS 3.60
Digital photo colorimeter	Medizinn, India	-
CO_2_ incubator with an orbital shaker	Eltek orbital shaker incubator	6161-LS
Polyvinyl chloride (PVC) sheet	Sigma-Aldrich	-
Copper tape	Robu Electronics	16045
CHI instrument	CH Instruments, Inc. 3700 Tennison Hill Drive Austin, USA	1030E
PMMA	Dali Electronics	-
Oxygen plasma	Femto Science, South Korea	-
Portable potentiostat (Sensit Smart)	Palmsens	-

## Step-by-step method details

### Fabrication of screen printed electrodes

**Timing: 3–4 h**

This section describes the fabrication of a three-electrode system using the screen-printing method. The step-by-step procedure is mentioned below:

1. Adhere to the polyvinyl chloride (45 × 300 cm) sheet on the glass slide.
2. Build a mask of the requisite design (Width of 1000 μm, spacing of 350 μm, thickness of 50 μm) using a commercial CO_2_ laser on a polyvinyl chloride (PVC) sheet. The design is shown in Figure 1.

**Figure 1:**
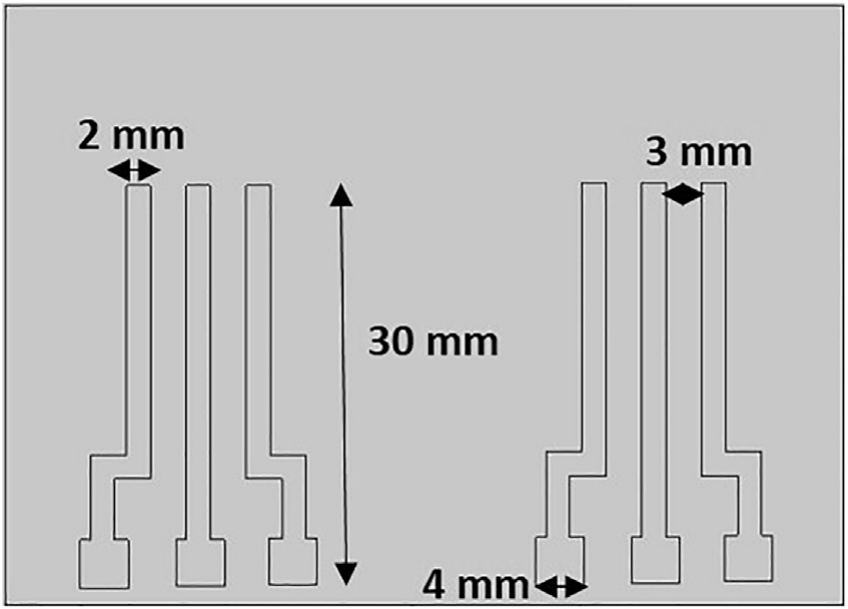
Pattern describing the design of the three-electrode system
3. Spread the carbon ink (The volume chosen here is 300 μL as the surface area of electrodes is 0.2 mm^2^) with a squeegee.
4. Afterward, set the prepared mask aside in the laboratory hot air oven for 40 min at 70°C.
5. Remove the PVC film after the ink is dried, keeping the three-electrode system over the glass slide. The details of the fabrication steps are mentioned in Figure 2.

**Figure 2:**
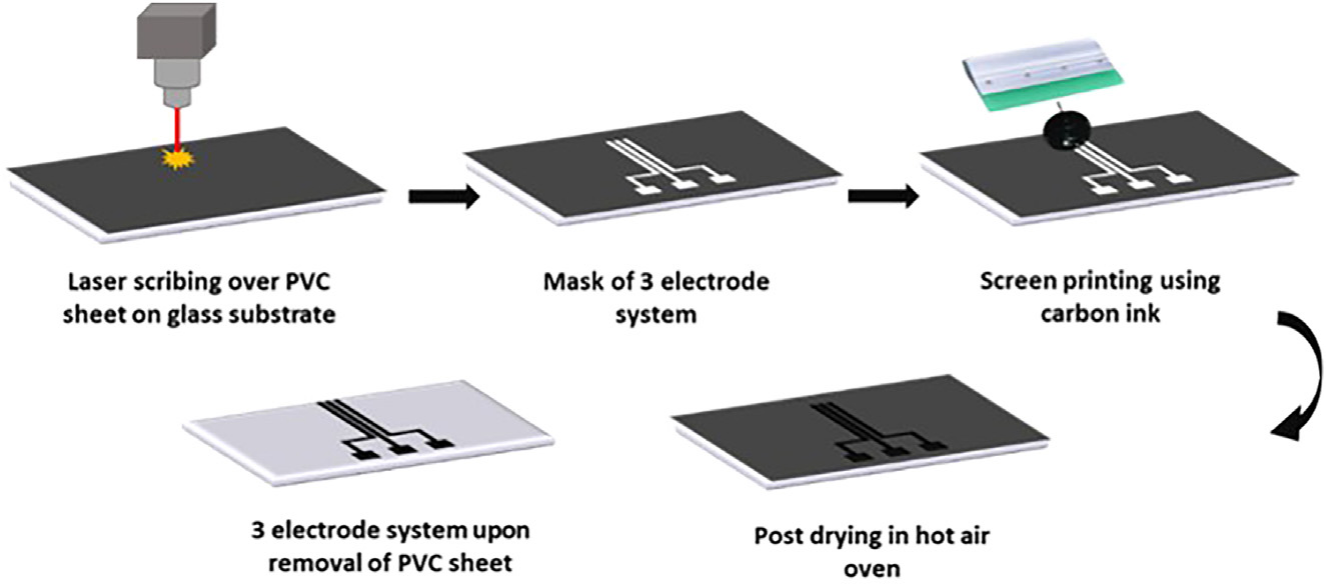
Schematic describing the step-by-step process showing how the three-electrode system is fabricated using screen printing
6. For electrochemical detection of bacterial growth, fabricate the three-electrode system.
  a. Treat one of the three electrodes with silver/silver chloride paste.
  b. Modify the working electrode with MWCNT, and dry it for 20 min at room temperature (20°C–25°C).

***Note:*** The carbon ink should be spread uniformly over the electrode surface.

### Characterization of a fabricated three-electrode system

**Timing: 2 h**

The initial electrochemical ferricyanide detection is performed on the developed electrodes to verify the effectiveness of the developed electrodes.

7. Prepare 5mM potassium ferricyanide in 1mM potassium chloride.
8. Use a pipette to drop the prepared solution (20 μL) onto the electrode surface.
9. Perform cyclic Voltammetry in a CHI instrument to measure the transfer of electrons at a scan rate of 0.05 V/s.
  a. This indicates redox behavior within the potential range of -0.7 and +0.7 V.
  b. Cyclic voltammetry responses are shown in Figure 3.

**Figure 3:**
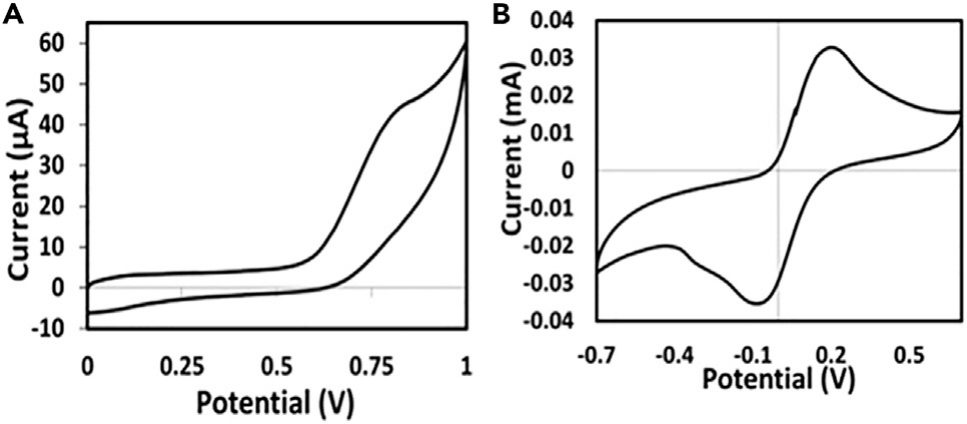
Cyclic voltammograms for plain LB media and potassium ferricyanide Cyclic voltammograms representing (A) Plain LB media in the absence of bacteria within a potential of 0–1 and (B) Potassium Ferricyanide within a potential of -0.7 to + 0.7.
10. Evaluate the electrodes for ordinary LB broth at potentials ranging from 0 to 1, and proper media peak is found at a potential of about 0.8 V.

### Fabrication of laser-induced graphene heater

**Timing: 1 h**

The LIG film is developed as a heating material for bacterial culturing.

**Figure 4:**
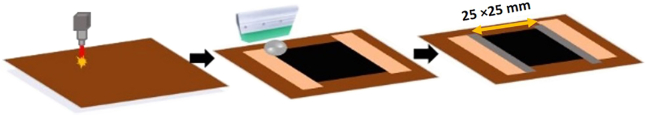
Schematic representing the steps for fabrication of the LIG heater
14. Next, 2.3V of potential is applied to the buck-boost converter, which provides a temperature of 37°C.

***Note:*** The temperature of the LIG heater should be maintained at 37°C. The fluctuation in temperature could cause cell death.

11. Stick a polyimide sheet of 10 mils × 920 mm thickness to the acrylic substrate using tape.
  a. Carved the polyimide sheet with a CO_2_ laser to produce laser-induced graphene.
12. The LIG heater provides a temperature of 37°C, which helps bacteria grow. The temperature is optimized
  a. By giving different voltages
  b. Capturing the image in a thermal camera.
13. Once LIG pattern is formed over the polyamide sheet,
  a. apply the silver paste
  b. Stick the copper tape to provide electrical contact. The Schematic is shown in Figure 4.

### Fabrication of microfluidic device and its integration]

**Timing:5–8 h**

This section describes the step-wise procedure for the fabrication of PDMS based microfluidic device^8^ and its integration on the three-electrode system.

15. Fabricate a microfluidic device using soft lithography.^9^
16. Prepare a design of the desired pattern in AutoCAD software. The pattern of the PDMS device is shown in Figure 5.

**Figure 5:**
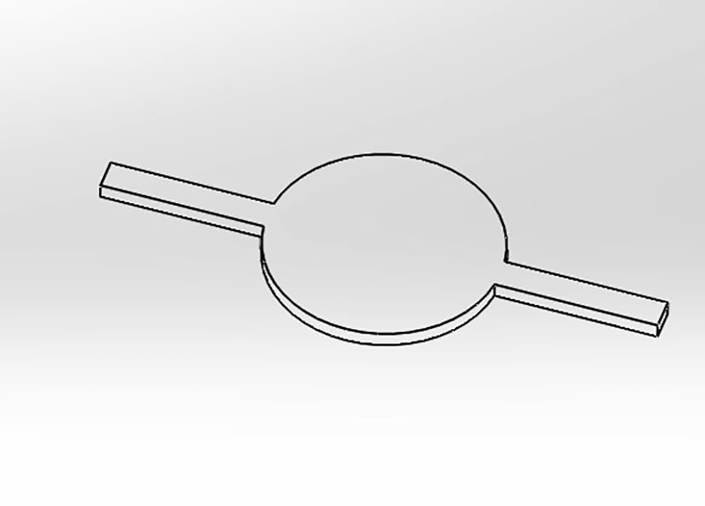
The pattern describing the design of the PDMS device
17. Cut the pattern on a PMMA (polymethylmethacrylate) using a CO2 laser.
  a. Adhere it to a glass substrate.
  b. Place in a mold per the well-established protocol.
18. Prepare PDMS by mixing the silicone elastomer with the curing agent in a ratio of 10:1 and is thoroughly mixed.
19. Degases the mixture in a desiccator, pour over the mold, then place in an oven at 65°C for curing.
20. Punch the required holes on the inlet and outlet of the PDMS slab after Post curing
  a. Use blunt needles to make holes.
  b. Bond the glass slide with screen-printed electrodes by treating the surfaces in the presence of oxygen plasma.

### Bacteria culture in the device

**Timing: 1–2days**

***Note:*** Prepare Luria Bertani (LB) containing 10 grams of powdered LB broth in 250 mL of distilled water.

21. A culture of E. coli (DH5) is prepared in a Bacterial suspension of OD 1 by adding 0.2 mL of the bacteria to 20 mL of the media. The schematic is shown in Figure 6.

**Figure 6:**
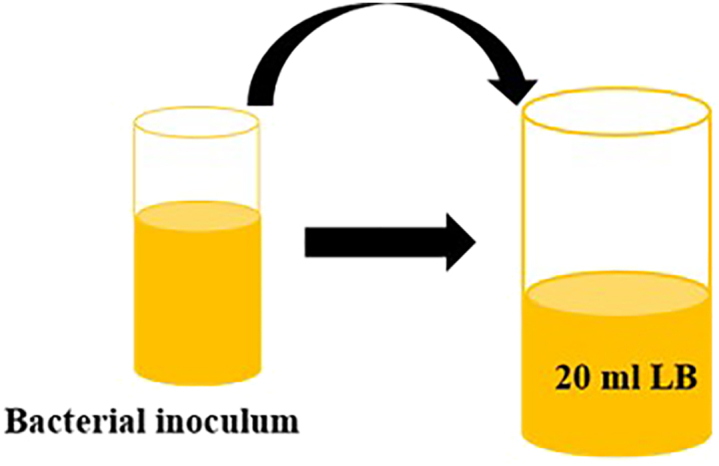
Schematic representing preparation procedure for bacterial culture
22. After the addition, the sample is well mixed (OD 0.8) and injected into a microfluidic reservoir using a syringe, and the temperature is kept at 36°C using a LIG heater. The response is also electrochemically detected.

### Electrochemical detection of bacteria

**Timing: 1–2 days**

Electrochemical detection uses i) cyclic voltammetry (CV) and ii) chronoamperometry (CA) methods.

23. The inoculum of the *E. coli* is introduced into the microfluidic reservoir and the conventional incubator for 12 h, and the result is compared.^10^
24. The three electrodes are connected to the portable potentiostat (Sensit Smart, Current ranges 100 nA–5 mA, Potential range -1.7 to +2 V), and the CV technique is selected.
25. Next, the software sets parameters like potential 1 to -1, No. of cycle-4, and scan rate- 0.05V.
26. Then, the run starts by clicking on the play button.
27. After completing the four-cycle, the obtained data will be extracted, and the graphs will be plotted.
28. Similarly, the potential is fixed at 0.8V for 120 min for chronoamperometry, and chronoamperometry is performed for different concentrations.
29. The responses are recorded and compared with a conventional incubator.

## Expected outcomes

Two electrochemical methods, Cyclic Voltammetry,^11^ and Chrono Amperometry^12^ are used to identify the presence of bacteria in the device.

Both approaches record the peak response, which detects the growth of bacteria in the device by plotting the calibration graph.

**Figure 7:**
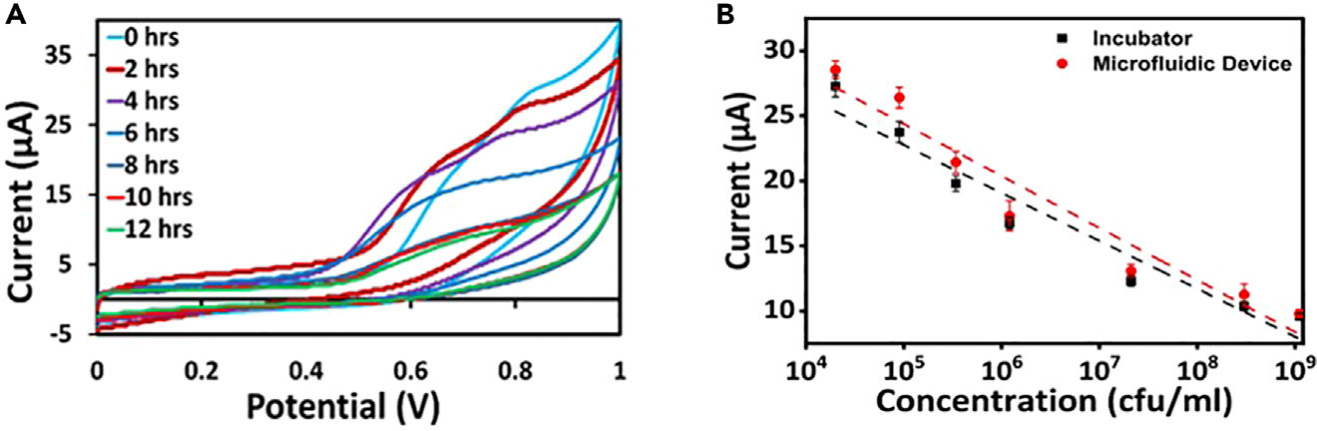
Cyclic voltammogram and calibration plots (A) Cyclic voltammogram recorded for 12 h corresponding to different concentrations of bacteria ranging from 2 × 10^4^ to 1.1 × 10^9^ CFU/mL in a Microfluidic device. (B) Calibration plots with error bars for samples incubated in a conventional and developed device.

Initially, CV is performed for 12 h in the device and conventional incubator to check the growth of bacteria. And the current is measured at intervals of 2 h. The current value obtained is analyzed and compared with the conventional method. Figure 7 represents the plot for the bacteria culture carried out in the microfluidic device.^13^ The concentration of bacteria increases over time in both the incubator and the microfluidic device.

In addition to a CV, the chronoamperometry approach is used in traditional and microfluidic devices to assess the characteristics of curves with a rise in bacterial concentration.^14^

**Figure 8:**
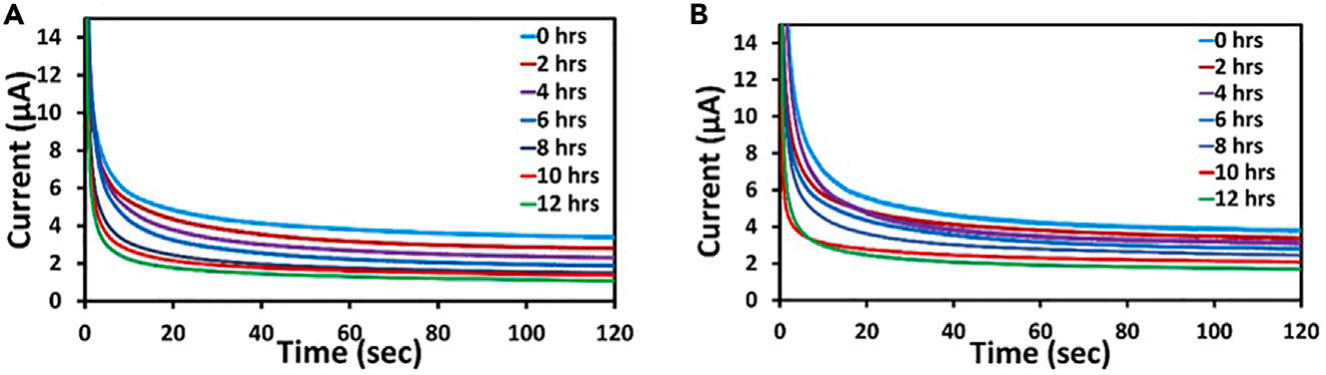
Chronoamperometric curves Chronoamperometric curves recorded for 12 h corresponding to different concentrations of bacteria ranging from 2 × 10^4^ to 1.1 × 10^9^ CFU/mL in (A) Conventional incubator and (B) Microfluidic device.

Chronoamperometry generally records the current about the time at a fixed potential.^15^ The peak is noted at approximately 0.8 V on the CV plots. One hundred 20 s are spent conducting each experiment at a constant potential voltage of 800 mV Figures 8A and 8B demonstrate that the current consistently decreases as concentration increases, similar to CV graphs.

## Limitations

The vitality of the bacteria culture from the device can be further increased by changing the device to have a shaking module duplicating the condition of the conventional incubator. Different E. Coli species cannot be differentiated in the device.

## Troubleshooting

### Problem 1

Device fabrication & electrode modification is a manual process hence, the reproducibility for multiple devices is challenging and depends on manual skill (related to step 6).

### Potential solution

Using an automated device fabrication approach, we can achieve excellent reproducibility.

### Problem 2

How is the movement of the bacteria addressed? What if they move out of the range of the electrodes? (related to step 24).

### Potential solution

The movement of bacteria can address by optimizing the design dimension. If they move the out of range, electrode saturation may be happening.

### Problem 3

Are bacteria attracted to electrodes? How would this affect the results, or how could it be addressed?

### Potential solution

Bacteria are not attracted to the electrode. However, adsorption may happen due to electrostatic forces.

### Problem 4

Bonding the PDMS channel to the SPE electrode is difficult and leads to leakage during the experiment (related to step 20).

### Potential solution

Minimizing the thickness of the electrode and exposing it for more time in plasma is helpful in perfect bonding. The optimized thickness of the three-electrode system is 50 μm, and the optimal time for plasma treatment is 1 min.

## Resource availability

### Lead contact

The lead contact, Sanket Goel, can be reached at (sgoel@hyderbad.bits-pilani.ac.in) and will reply to any questions or requests for resources.

### Materials availability

This protocol does not generate unique reagents or material.

## Data Availability

The lead contact will, upon request, provide the data provided in this research. The lead contact will provide any further information needed to refocus the data given in this study.
